# Modeling of Mutational Events in the Evolution of Viruses

**DOI:** 10.3390/v11050418

**Published:** 2019-05-05

**Authors:** Akhtar Ali, Ulrich Melcher

**Affiliations:** 1 Department of Biological Sciences, University of Tulsa, Tulsa, OK 74104, USA; akhtar-ali@utulsa.edu; 2 Department of Biochemistry & Molecular Biology, Oklahoma State University, Stillwater, OK 74078-3035, USA

**Keywords:** virus–host co-divergence, endogenous viral elements, virus networks, speciation

## Abstract

Diverse studies of viral evolution have led to the recognition that the evolutionary rates of viral taxa observed are dependent on the time scale being investigated—with short-term studies giving fast substitution rates, and orders of magnitude lower rates for deep calibrations. Although each of these factors may contribute to this time dependent rate phenomenon, a more fundamental cause should be considered. We sought to test computationally whether the basic phenomena of virus evolution (mutation, replication, and selection) can explain the relationships between the evolutionary and phylogenetic distances. We tested, by computational inference, the hypothesis that the phylogenetic distances between the pairs of sequences are functions of the evolutionary path lengths between them. A Basic simulation revealed that the relationship between simulated genetic and mutational distances is non-linear, and can be consistent with different rates of nucleotide substitution at different depths of branches in phylogenetic trees.

## 1. Virus Evolution Introduction

Viruses are often thought of as agents that have evolved to threaten humans and other manifestations of life. This view is strengthened by the impression that many disease epidemics (of humans, other animals, and plants) are of viral instigation. Nevertheless, the relationships between viruses and their hosts cover the full range, from mutualists or symbionts to commensals to pathogens [1,2,3,4]. As life forms have evolved, so too have the viruses associated with them. These observations generate the hypothesis that many viruses have co-diverged with their hosts during evolution. Here, we discuss ways to reconcile conflicting claims of high mutation rates in short-term evolution studies with low nucleotide substitution rates at longer time scales.

Dating the emergence of viral lineages is important for understanding the epidemiology of viral outbreaks [5]. Dating has been complicated by the observation that the rates of substitution of nucleotide and/or amino acid residues in viral genes or gene products, respectively, are dependent on the time scales of the events being dated [6,7,8]. A variety of approaches to dating phylogenetic trees, covering different time periods, have been taken. In one approach, the rates of nucleotide substitution in viral genomes have been determined in prospective experiments. In these, hosts are inoculated with a genetically homogeneous viral genome, and hosts are then sampled at various times to determine how many nucleotides have changed [9,10,11]—a process whose usefulness is debated [12]. The typical substitution frequencies observed by such techniques are 10^−3^ to 10^−4^ substitutions per residue per year.

Phylogenetic inference methods are based on comparisons of sequence differences among contemporaneous viral isolates. In heterochronous sampling, the sequences obtained from viruses isolated at different times [13,14,15,16] provide calibration points for phylogenetic comparisons. Specimens from more distant times [13] occasionally yield additional sequences that can be used to calibrate phylogenies [17,18,19]. Similarly, paleo-sequences, or endogenous viral elements (EVEs) [20], can also be used in the calibration of trees. The fact that phylogenetic trees, for numerous viral taxa, resemble, in topology and branch lengths, the trees constructed for their predominant hosts, argues for the co-divergence of nucleotide sequences of the host and virus during the evolution of host lineages [21], and provides a further calibration approach.

### Time-Dependent Rate Phenomena

The integration of results from these diverse studies led to the recognition that the rates observed are dependent on the time scale being investigated—with short-term studies giving fast substitution rates, with orders of magnitude having lower rates for deep calibrations [20]. The current issue is how the number of evolutionary steps needed to evolve from an ancestor is related to the phylogenetic distance between the tip and ancestor. Power law and exponential relationships have been proposed and tested for the relationship of rates to time spans [22]. Artefacts and biases have been proposed as explanations for the discrepancies [12]. Various ways of correcting for time-dependent rate phenomena (TDRP) [8] have been proposed, including different scales for different biological epochs [23,24], avoiding sequencing errors, correcting for tree imbalances [25], and a better treatment of the influence of rate heterogeneity among sites. Accurate correction requires an understanding of the root causes of TDRP.

A variety of factors that may produce TDRP have been suggested. These include, hypermutation, recombination, and variations in selective forces [26]. Although each of these factors may contribute to the TDRP, a more fundamental cause should be considered. We sought to test computationally whether the basic phenomena of virus evolution (mutation, replication, and selection) can explain the relationships between the evolutionary and phylogenetic distances. Specifically, we aimed to test the hypothesis that the phylogenetic distances between the pairs of sequences are functions of the evolutionary path lengths between them.

## 2. Materials and Methods

To test the hypothesis, we defined the phylogenetic distance as the number of loci (sequence positions) that differ between the two sequences and evolutionary distances, as the number of modeled mutations used to transition between the two sequences, given the mutation, replication, and selection processes. We devised a simple program (Mevolve.c4d; Supplementary Material) to generate evolutionary distances using a network similar to the neutral network of Manrubia and Cuesta [27], except that we focused on non-neutral mutations. Genomes were represented as strings of an arbitrary length of 12, representing variable positions in the sequence. Each position in the string is diallelic [28], having just two states, “0” and “1”, that can mutate from one to the other. A “1” in a position contributes a positive effect on the fitness of the virus in a particular environment, the latter assumed to be held constant. The program incorporates mutation, replication, and selection in cycles. Inputs are a “seed” string and a target string. The initial homogeneous seed population of size ten is subjected to the mutation of a single digit per string—the position of the mutation being chosen randomly.

After this mutation step, the population is allowed to expand to 100 members by replication. The number of offspring produced by each string is determined by the fitness of the string (determined by the number of “1” in the chain, the string’s fitness index). To implement the selection, the population size is then lowered to the original ten by a bottleneck, in which ten random selections of pairs are compared by fitness index values, keeping the more fit for the next round of 10 strings. The process of mutation, replication, and selection is repeated for as many rounds as desired (a variable set by the user). The calculation is continued for as many times as needed to produce the target string of one of the ten selected population members. When the target string is obtained, the routine reports the number of repetitions (cycles) used. The routine was executed in Chipmunk Basic [29].

## 3. Results

**Figure 1:**
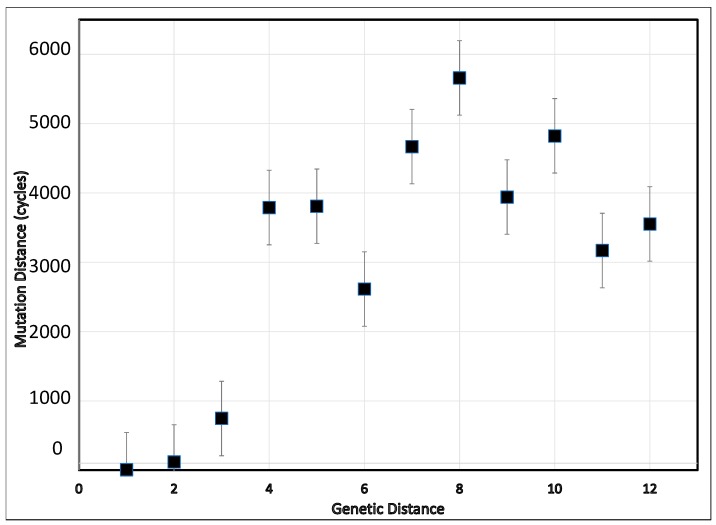
For hypothetical genomes containing 12 differentiating loci, the relation between the genetic distance (number of loci at which the members of a pair being analyzed differ) and mutational distance (number of mutational events needed to convert one member of the pair into the other) in a simulation of cycles of repeated random mutation, replication, and competition for 12 loci, allowing for only two alternative states, modeled as ”0” and “1”. Lines extending from the markers represent the standard error of five repetitions of each possible seed string in conversion to “111111111111”.

The preliminary examination of the simulation script tested the effect of the relative order of string elements. It established that varying the positions of the “0” and “1” elements of the seed and target strings was without effect on the numbers of simulation cycles. The mean numbers of evolutionary cycles needed to increase the fitness index from a given level to the various higher fitness levels are illustrated in Figure 1. The simulation revealed that the relationship between the simulated genetic and mutational distances is non-linear, and can be consistent with different rates of nucleotide substitution observed at different depths of branches in phylogenetic trees. As expected because of the random calculation steps integral to the simulation script, considerable variation in the extent of mutation distances (computational cycles) occurred, as reflected in the substantial standard errors. Despite these large standard errors for numbers, the mutation distance increased as the genetic distance increased from 1 to 5. Also, as expected, the mutation distance increased with the genetic distances. Not unexpectedly, using targets with more than six negative alleles (genetic distance = 6) resulted in the highest number of computational cycles to reach a string consisting of all positive alleles. Considering transitioning from a distance of two viral substitutions to eight, such a change was accompanied by close to 6000 cycles of mutation, replication, and selection. Approximately 3000 cycles underlie a genetic distance leap of three substitutions (from two to five).

## 4. Discussion

**Figure 2:**
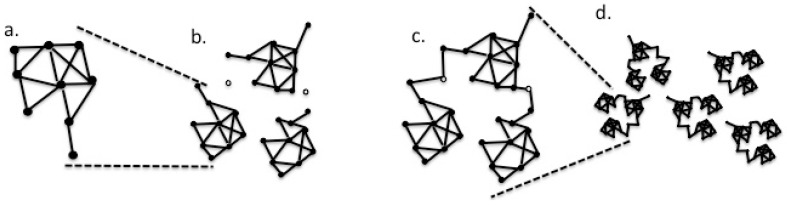
Diagram of the network concept of the viral evolution, where solid nodes represent viable viral genome sequences, and edges represent single nucleotide changes that connect the nodes. Open nodes represent sequences that are conditionally viable, namely: (**a**) basic single module, perhaps a viral isolate; (**b**) three such modules near one another; and (**c**) modules of (**b**) now connected through the formation of a supermodule by joining at conditional nodes. The supermodule may be equivalent to a viral species; (**d**) a potentially higher-level module composed of supermodules, potentially forming a viral genus. The network should form a module with a high connectivity. A plot of the frequencies of the node connectivity values (measured as k, degree, which are determined by the number of edges attached to a node) should follow a Poisson-like distribution [30]. There will be multiple paths connecting many pairs of nodes to each other, yet all of the nodes must be connected to the network by at least one edge.

Viral populations are often thought of as quasispecies. They are networks consisting of viral genome segments as edges, connected by nodes at the edge ends. These are capable of mutational changes. In the present context, the mutation of a node residue to one that no longer interacts with a partner closes the edge to substitution. Conversely, a change in another network node can open an interaction with another network module (Figure 2).

The concept of a network of functional loci connected by mutational paths, as playing a significant role in the evolution of viruses in host environments, may help to imagine viral sequence space as a random network in which each node represents a viable viral genome sequence. Each node is directly connected to all of the other nodes by a bidirectional edge, so that it can reach by a single nucleotide mutation (Figure 2a). Conversely, a change in another network node can open an interaction with another species network. In the computational simulation, the recorded substitutions that do not contribute to the genetic distance may correspond to the networks of changes that open or close the gates connecting the submodules of the population.

**Figure 3:**
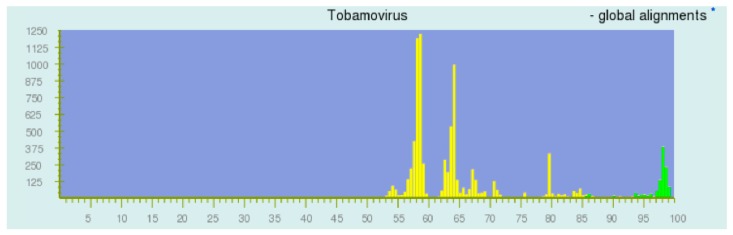
Pairwise sequence comparison (PASC) display of the distance relationships among the *Tobamovirus* sequences (Screenshot, March 2015). A histogram of the numbers of sequence pairs yielding binned similarity values. Green bars are as a result of within species comparisons, and the yellow bars are between members of different species.

The appropriateness of the network model is supported by the success of pairwise sequence comparison (PASC) [1,31,32,33,34,35,36] and the DEmARC analysis [37]. PASC is a web-based tool (http://www.ncbi.nlm.nih.gov/sutils/pasc) originally devised to both support the virus taxonomy and to facilitate it. A PASC plot (Figure 3) displays the frequencies of the sequence pair similarities in the bins of the nucleotide similarities for all pairs of taxa being considered, and thus represents the average connectivity within a network cluster.

These frequencies, for many collections of taxa, exhibit a series of peaks and valleys. Each peak has a Poisson-like distribution, as expected from the network theory. The peaks are interpreted as substitution distances for a particular taxonomic level (within strain, within species, within genus, within family, etc.). In this way, pairs can be assigned as members of the same strain of a species, or as different strains of the same species, or as different species of the same genus, and so on, depending on the peak order. Such a plot is predicted by the network theory, where each peak corresponds to a module of related sequences with other modules of the same divergence.

Recall that nodes are defined as viable genome sequences, genomes whose fitness is greater than some arbitrary value. “Viable” or “fit” are used here to characterize a genome molecule that, when placed in a given environment, will generate multiple copies of itself (*R_o_* > 1). Some nucleotide residues in viral genomes can mutate without major effects on the genome fitness. Groupings of nodes interconnected by a high density of edges, called modules, can be imagined as describing a strain of a virus. A module for strain A of a virus will be distinct from a module formed by strain B sequences, with few, if any, edges joining the two modules.

There will be fewer nodes at the periphery of a module. Connections from strain A outlier nodes to similar nodes of strain B may exist. These connections provide an evolutionary path between strains A and B. Such connections likely will require further mutational changes (node connections). Over time, the taxa will wander their landscape networks. Occasionally, one will wander to the vicinity of a node shared with another module, representing another taxon or potential taxon—a boundary.

The addition of further strain modules will result in a supermodule, depicting the possible evolutionary paths within a viral species. At a still larger scale, the species supermodules may connect to create genus super-supermodules. The process is extensible to larger and larger scales. Thus, the structure of the connectivity diagrams is independent of scale.

The simple computational strategy developed here to address the explanation of the TDR can also be modified to model events, other than replication, mutation, and selection, that affect evolution. Such events can include new hosts, environmental changes, host defenses, and agricultural practice. Introducing into models modified 12mer strings that are defined to signal path closure, or new options for a particular host environment, could reveal new pathways of evolution.

## Supplementary Materials

The following are available online: https://www.mdpi.com/1999-4915/11/5/418/s1 (Mevolve.c4d).
